# Combined effects of feed-grade urea and a urease inhibitor on growth performance, digestibility, blood biochemical indicators, and rumen environment in beef cattle

**DOI:** 10.3389/fvets.2025.1589125

**Published:** 2025-05-06

**Authors:** Yuan Zhou, Xuerong Song, Dabo Fu, Lizbeth E. Robles-Jimenez, Manuel Gonzalez-Ronquillo, Navid Ghavipanje

**Affiliations:** ^1^Wuhan Academy of Agriculture Sciences, Wuhan, Hubei, China; ^2^Wuhan Sunhy Biology Co., Ltd., Hubei, China; ^3^Instituto De Investigaciones En Ecosistemas Y Sustentabilidad, Universidad Nacional Autonoma de Mexico, Morelia, Michoacán, Mexico; ^4^Facultad de Medicina Veterinaria y Zootecnia, Universidad Autonoma del Estado de Mexico, Toluca, Estado de México, Mexico; ^5^Department of Animal Science, Faculty of Agriculture, University of Birjand, Birjand, Iran; ^6^South Khorasan Agricultural and Natural Resources Research and Education Center, Agricultural Research, Education and Extension Organization (AREEO), Birjand, Iran

**Keywords:** ammonia nitrogen, non-protein nitrogen, rumen fermentation, urea, sustainability

## Abstract

This study evaluated the combined effects of feed-grade urea (FGU) and a urease inhibitor (HyUrit) as a partial replacement for vegetable protein sources, specifically soybean meal (SBM), in beef cattle diets. The outcomes assessed included growth performance, nutrient digestibility, blood parameters, and the rumen environment. A total of 30 Simmental bulls [average initial body weight (IBW): 445.67 ± 26.48 kg] were assigned to a completely randomized design (CRD) and fed diets containing FGU supplemented with 0, 5, 10, 15%, or 20% of a urea inhibitor (HU) on a dry matter (DM) basis. There were no significant dietary effects on IBW (*p* = 0.843), final body weight (FBW; *p* = 0.912), or average daily gain (ADG; *p* = 0.372). Similarly, the intakes of DM (*p* = 0.906), organic matter (OM; *p* = 0.939), crude protein (CP; *p* = 0.898), neutral detergent fiber (NDF; *p* = 0.565), and acid detergent fiber (ADF; *p* = 0.616) were not affected by dietary treatments. However, supplementation with HU at 10, 15, and 20% significantly improved the digestibility of OM (*p* = 0.001), CP (*p* = 0.030), NDF (*p* = 0.001), and ADF (*p* = 0.001) compared to the FGU group; the digestibility of DM (*p* = 0.651) remained unaffected. Neither nitrogen intake (g/d, *p* = 0.898; g/LW^0.75^, *p* = 0.707) nor nitrogen balance (g/d, *p* = 0.614; g/LW^0.75^, *p* = 0.755) was significantly affected by different levels of urease inhibitor supplementation. Additionally, dietary treatment did not significantly affect the plasma concentrations of alkaline phosphatase (ALP; *p* = 0.319), albumin (*p* = 0.080), insulin (*p* = 0.217), or insulin-like growth factor-I (IGF-I; *p* = 0.094) in beef cattle. However, the activities of aspartate aminotransferase (AST) and alanine aminotransferase (ALT) were significantly increased in the HU15 group (15% HyUrit), with *p*-values of 0.002 and 0.011, respectively. In addition, total plasma protein concentration was significantly lower in the HU20 group compared to both the Ctrl and HU5 (5% HyUrit) groups (*p* = 0.012). *In vitro,* ammonia nitrogen (N-NH_3_) production at 0 h (*p* = 0.0001), 0.5 h (*p* = 0.0009), and 2 h (*p* = 0.0001) was higher in both the FGU- and HyUrit-containing groups than in the Ctrl (SBM) group. Overall, the lower cost of FGU may justify its partial replacement of true protein meals in beef cattle diets without compromising growth performance or animal health, especially in cases where FGU is consumed as a single dose rather than through a total mixed ration (TMR) at the HU20 level (20% HyUrit), thereby mitigating the risk of alkalosis.

## 1 Introduction

Ruminants convert inedible resources into high-quality protein (meat and milk), which is vital for human nutrition (1). The growing population, increasing income levels, and urbanization have led to a 70% increase in the demand for animal protein from 2005 to 2050.

In ruminant nutrition, dietary protein serves a crucial role in supplying amino acids (AAs) and nitrogen (N), which fuels the production of microbial crude protein (MCP) in the rumen (2). MCP serves as the primary source of crude protein (CP) that flows into the small intestine, and when combined with dietary rumen undegradable protein (RUP), it forms the metabolizable protein (MP) that is digested and absorbed in the small intestine to satisfy the AAs requirements of ruminants (3). However, protein-rich feedstuffs like soybean meal (SBM) are primarily associated with high costs and environmental footprints, leading researchers to seek out alternatives. Consequently, incorporating non-protein nitrogen (NPN) into ruminant diets has emerged as a viable strategy that not only boosts MCP and reduces feeding costs but also mitigates food-feed-fuel competition and lowers both water and carbon footprints (4, 5).

Despite decades of research, non-protein nitrogen (NPN) continues to be a topic of great interest and debate in ruminant nutrition (6–8). Feed-grade urea (FGU) is the most widely used NPN source among various NPN compounds; however, its application in ruminant diets is limited due to its rapid hydrolysis to ammonia-N (NH_3_) in the rumen, which results in asynchrony between NH_3_ and fermentable energy, adversely affecting microbial crude protein (MCP) synthesis and outflow (5, 9). Additionally, the rapid hydrolysis of FGU in the rumen can decrease nitrogen (N) utilization, increase N excretion, elevate blood NH_3_ levels, and raise the risk of NH_3_ toxicity (10, 11). Different coated ureas or slow-release urea products have been developed to consistently supply ammonia in the rumen, avoiding the potential toxicity associated with FGU (4, 12). Moreover, urease inhibitors have emerged as another mitigation tool that controls the rate of urea degradation and NH_3_ release in the rumen (4, 8, 13). Urease catalyzes the hydrolysis of urea into ammonia and carbon dioxide. It is widely distributed among various bacteria, enabling survival in diverse ecological niches (14). A urease inhibitor can effectively reduce the ureolytic activity of rumen bacteria, making it a viable method to enhance the utilization of both dietary and endogenous urea by the ruminal microbiota and ruminant animals (15). It has been demonstrated that urease is composed of apo-urease and accessory proteins, including UreD (UreH), UreE, UreF, and UreG. Apo-urease is commonly targeted for the design of urease inhibitors, which either compete with the active sites of urease or mimic its substrate, urea (16). The literature highlights that urease inhibitors are significant for improving animal production and environmental nitrogen conservation (15).

Feeding strategies that aim to utilize FGU as a replacement for conventional protein (i.e., SBM) must first be achieved without compromising rumen function. Future studies are warranted to explore more suitable nitrogen sources as substitutes for protein feed. Therefore, the present study aimed to investigate the feasibility of using FGU (in place of SBM) with the addition of different levels of a urease inhibitor (HyUrit) in beef diets. The response variables included nutrient intake and digestibility, growth performance, blood parameters, and ruminal fermentation. We hypothesize that the combined effects of FGU and the urease inhibitor can effectively improve beef cattle production due to their impact on rumen functionality and potentially lower environmental impact.

## 2 Materials and methods

### 2.1 Site study and ethics statement

This study was conducted at the Wuhan Academy of Agricultural Sciences. The experimental implementation and procedure were approved by the Professional Committee on Standardization of Experimental Animals of the Wuhan Academy of Agricultural Sciences (WHNKYAMO-202207040), and all animal care was conducted in accordance with relevant institutional, national, and international guidelines and regulations.

### 2.2 Animals, diets, and experimental design

The study involved 30 Simmental bulls with a mean initial body weight (IBW) of 445.67 ± 26.48 kg [mean ± standard deviation (SD)]. The bulls were randomly assigned to receive one of six dietary treatments (n = five per diet) over a 38-day experimental period (10 days of adaptation and 28 days of experimental treatment). The diets, consisting of 70% silage and 30% concentrate, were formulated to be isoenergetic (2.38 Mcal ME/kg DM) and isoproteic (13.5% CP) according to the Beef Cattle Feed Standard (NY/T815-2004). The dietary treatments were supplemented with different nitrogen sources as follows: (1) soybean meal (SBM) as the reference protein source (Ctrl); (2) feed-grade urea with 0% HyUrit (FGU); (3) FGU with 5% HyUrit (HU5); (4) FGU with 10% HyUrit (HU10); (5) FGU with 15% HyUrit (HU15); (6) FGU with 20% HyUrit (HU20). HyUrit (Sunhy Trading Co., Ltd., Wuhan, China) is a commercial feed-grade urease inhibitor containing 10% acetohydroxamic acid as the active ingredient. The animals were housed in individual stalls (2.40 × 1.45 m) and were fed twice a day, at 08:00 and 18:00, with each animal serving as an experimental unit. In addition, the bulls in all experimental groups received water ad libitum. The ingredients and chemical composition of the experimental diets are presented in Table 1. Notably, all animal handling and laboratory staff were blinded to the study diets.

**Table 1:** Ingredients and chemical composition of diets fed to beef cattle containing feed-grade urea (FGU) and urease inhibitor (HyUrit).

Items	Diets					
	Ctrl	FGU	HU5	HU10	HU15	HU20
Corn grain, g/kg	104	155	154.55	154.3	153.95	153.6
Soybean meal, g/kg	78	24	24	24	24	24
Corn silage, g/kg	260	258	258	258	258	258
Yellow corn silage, g/kg	416	414	414	414	414	414
Haulm, g/kg	52	52	52	52	52	52
Wheat bran, g/kg	70	70	70	70	70	70
Vitamin-mineral premix^1^, g/kg	20	20	20	20	20	20
Urea, g/kg	0	7	7	7	7	7
HyUrit, g/kg	0	0	0.35	0.7	1.05	1.4
Chemical composition						
DM, g/kg	545.89	547.80	547.80	547.80	547.80	547.80
CP, g/kg	134.7	136.0	136.0	136.0	136.0	136.0
peNDF, g/kg	276.4	270.5	270.5	270.5	270.5	270.5
NDF, g/kg	406.1	391.8	391.8	391.8	391.8	391.8
ADF, g/kg	252.5	244.3	244.3	244.3	244.3	244.3
ME, Mcal/kg	2.39	2.36	2.36	2.36	2.36	2.36

^1^Composed of zinc sulfate (43.5%), copper sulfate (15.8%), manganous sulfate (22.4%), EDDI 80 (0.22%), selenium (7.7%), cobalt carbonate (0.12%), vitamin A (2.6%), vitamin D (0.26%), and vitamin E (7.25%).

### 2.3 Data collection and measurements

#### 2.3.1 Animal performance

Individual body weights were measured at the start and end of the experimental period after a 16-h fasting period, prior to the first-morning feeding, using a Pioneer™ Precision High Capacity (PR224ZHE, Ohaus Instrument Co., Ltd., Changzhou, China). Average daily gain (ADG) and gain-to-feed ratio (G: F) were calculated by dividing body weight gain by the number of experimental days and total dry matter intake, respectively.

The amounts of feed offered and refused were measured daily to determine dry matter intake (DMI), but only data from the last 6 days were included in the statistical analysis. During the last 6 days of the experimental period (days 31–37), feces and refusals were collected daily, and 10% of the total fecal samples were frozen at −20°C for further analysis of digestibility and nitrogen balance. Approximately 200 g of fecal samples were collected from each bull’s rectum at 07:00 and 19:00, mixed with a 10% solution of sulfuric acid, and kept at −20°^C^ for later analysis. The samples of TMR and feces were thoroughly mixed, dried at 55°C for 72 h, and ground to pass through a 1 mm screen using a cutter mill (Beishengwei Experimental Instrument Co., Ltd., Changzhou, China).

#### 2.3.2 Ruminal fermentation

On day 38, approximately 100–200 mL of rumen fluid was collected 2 h after feeding from 30 beef cattle using a flexible oral tube via esophageal means, and the pH was determined immediately with a portable pH meter (ST3100/F, Zhejiang Scientific Equipment Co., Ltd., Zhejiang, China). An additional 100 mL of rumen fluid digesta was strained through two layers of cheesecloth and stored at −80°C for volatile fatty acid (VFA) analysis. VFA concentrations (μg/g) were analyzed using gas chromatography–mass spectrometry (GCMS) (ISQ 7610 Large Turbo, Thermo Scientific TM, Beijing, China). Column temperature: 100°C (5 min)-5/min-150 (0 min) − 30°C/min −240°C (30 min), flow velocity: 1 mL/s, split ratio: 75, carrier: helium, chromatographic column: TG WAX 30 m × 0.25 m, injector: 240, Mass Spectrometry: EI. Subsequently, two 25 mL aliquots of ruminal fluid were preserved in scintillation vials by adding 5 mL of 0.2 N HCl and stored at −20°C. Just prior to analysis, the samples were thawed and centrifuged (15,300 × g for 20 min at 4°C), and the supernatants were used to determine ammonia nitrogen (NH_3_-N, mg/dL) in a ruminal fluid using the protein-free filtrate method with the standard kit (Nanjing Jiancheng Bioengineering Institute) by a biochemical analyzer (Model 7,600; Hitachi, Tokyo, Japan).

#### 2.3.3 Blood sampling and analysis

On the last day, blood samples (approximately 10 mL) were collected from the jugular vein of each bull before morning feeding (0700 h) in tubes containing heparin sodium as an anticoagulant. Plasma samples were separated by centrifugation at 13,400 r/min for 30 min at 4°C, and the obtained supernatant was stored at −20°C for further analysis. Blood biochemical indicators were determined using the Chemiluminescence Immunoassay Analyzer (CL-6000i Mindray, Shenzhen, China) with applicable kits (Mindray, China): glucose (Glu, mmol/L) via the glucose oxidase method, blood urea nitrogen (BUN, mmol/L) via the UV glutamate dehydrogenase method, total protein (TP, g/L) via the biuret method, albumin (ALB, g/L) via the bromocresol green method, alanine aminotransferase (ALT, U/L) via the IFFC method, aspartate aminotransferase (AST, U/L) via the IFFC method, alkaline phosphatase (ALP, U/L) via the AMP buffer method, triglycerides (TG, mmol/L) via the oxidase method, and total cholesterol (TC, mmol/L) via the oxidase method. Both insulin-like growth factor-I (IGF-I, ng/mL) and insulin (m IU/L) were detected using the chemiluminescence method by a fully automatic chemiluminescence apparatus (AutoLumoA62000, Zhengzhou, China) with applicable kits (Mindray, China).

### 2.4 Laboratory analysis

All ingredients, TMR, orts, and fecal samples were dried in a forced-air oven at 60°C for 48 h and subsequently ground using a hammer mill (Willey, 2 mm Ø, Arthur H. Thomas, Philadelphia, PA, United States). The samples were analyzed in triplicate following AOAC methods (17) for DM (method no. 934.01), organic matter (OM, method no. 942.05), and ash (method no. GB/T 6438–2007). The CP content was determined in accordance with GB/T 6432–2018 using the Qianjian Fully Automatic Kjeldahl Nitrogen Determinator (KDN-19 K, Shanghai Qianjian Instruments Co., Ltd., Shanghai, China), and the ethereal extract (EE; Soxhlet System HT Analyzer; Foss Electric, Hillerød, Denmark; method no. GB/T 6433–2006). Acid detergent fiber (ADF) content was analyzed according to NY/T 1459–2022. The feces were used to determine nitrogen (which was assessed according to GB/T 6432–2018) using the Qianjian Fully Automatic Kjeldahl Nitrogen Determinator (KDN-19 K, Shanghai Qianjian Instruments Co., Ltd., Shanghai, China). cid-insoluble ash (AIA) content (18) in the rations and manure was determined according to GB/T 23742–2009, and apparent nutrient digestibility was calculated according to Lee and Hristov (19).

### 2.5 *In vitro* N-NH_3_ determination

Three beef steers [577 ± 48.75 kg body weight (BW); mean ± standard deviation (SD)] with a flexible rumen fistula were used as donors of rumen fluid inocula in the experiment. From 14 days prior to the trial, the animals were fed a total mixed ration (TMR) consisting of the control diet twice daily (0800 and 1,700 h), with ad libitum access to fresh water. The ruminal inocula were collected prior to the morning feeding (0600 h), mixed, strained through four layers of cheesecloth, and transported to the laboratory in a thermo-insulated container pre-filled with CO2.

Ammonia concentration and pH were determined in another four series of incubations in triplicate according to Theodorou et al. (20). Briefly, 0.800 g DM of all substrates was incubated in 125 mL calibrated glass bottles in triplicate for three incubation cycles with 100 mL of incubation medium containing 10 mL of filtered rumen fluid and 90 mL of a buffer solution. The flasks (nine flasks of each substrate) were placed vertically in a 39°C water bath, and three flasks without substrate were used as blanks. The solution in each flask was sampled at 1 mL at 0, 5, 1, 2, 4, and 8 h of incubation, acidified with 0.2 mL of 0.2 N HCl, and stored at −20°C. Just prior to analysis, the samples were thawed and centrifuged (15,300 × g for 20 min at 4°C), and the supernatants were used to determine ammonia (N-NH_3_, mg/dL) using the phenol-hypochlorite method (Lachat Method 18–107-06-1-A).

### 2.6 Statistical analysis

Data distribution and variance homogeneity were tested using PROC UNIVARIATE (SAS 9.4, SAS Institute Inc., Cary, NC, United Staes). The Shapiro–Wilk test assessed the normality of the data (*p* ≥ 0.05).

All data were statistically analyzed using a completely randomized design according to the following model:

Yijk=μ+αi+βj+εijk,

where y represents the measure of the experimental unit, μ denotes the overall mean, α*i* indissswcates the random effect of the fermentation run, β*j* signifies the fixed effect of the treatment, and ε*ijk* accounts for the residual error. A one-way analysis of covariance (ANCOVA) was conducted for the measurements, considering BW as a covariate. The Tukey–Kramer test was employed to compare least square means (LSM). Significance was established at *p* < 0.05, while 0.05 ≤ *p* ≤ 0.10 indicated a significant trend. Orthogonal polynomial contrasts were also utilized to assess the statistical significance of the linear and quadratic components of the treatments, evaluating specific trends in the data. The partial eta squared statistic (η^2^) was applied for parametric data, with small, medium, and large effects considered for η^2^ values of 0.01, 0.06, and 0.14, respectively.

## 3 Results

### 3.1 Growth performance

In the Shapiro–Wilk tests, values greater than *p* > 0.05 were obtained, so all data demonstrated normality. Data concerning production performance are reported in Table 2. There were no diet effects on IBW (*p* = 0.843), FBW (*p* = 0.912), ADG (*p* = 0.372), and FCR (*p* = 0.577). Additionally, the metabolic IBW (*p* = 0.844), FBW (*p* = 0.913), and ADG (*p* = 0.349) were not affected among treatments.

**Table 2:** Production performance of beef cattle fed diets containing feed-grade urea (FGU) supplemented with 0, 5, 10, 15, and 20% urease inhibitor (HyUrit).

Variable	Diets						SEM	Shapiro–Wilk	*p*-value			Effect size
	Ctrl	FGU	HU5	HU10	HU15	HU20			Treat	Lineal	Quadratic	
IBW, kg	445.20	432.40	446.80	444.92	449.20	441.20	12.507	0.418	0.843	0.737	0.208	0.382
FBW, kg	490.00	469.60	482.80	483.20	485.60	487.60	13.234	0.934	0.912	0.719	0.305	0.501
ADG, kg	1.24	1.32	1.28	1.21	1.30	1.65	0.152	0.803	0.372	0.894	0.597	0.429
Initial BW^0.75^	98.49	94.79	97.15	97.55	97.56	96.22	2.040	0.440	0.844	0.748	0.209	0.617
Final BW^0.75^	104.08	100.84	102.98	103.03	103.43	103.72	2.118	0.946	0.913	0.727	0.306	0.680
Average BW^0.75^	101.29	97.82	100.07	100.29	100.50	99.97	2.051	0.848	0.895	0.734	0.249	0.578
ADG ^0.75^	199.89	216.02	208.07	195.53	209.77	267.94	24.31	0.350	0.349	0.900	0.544	0.538
Feed gain ratio	7.75	7.57	7.50	7.97	7.16	5.91	0.838	0.850	0.577	0.854	0.784	0.687
Feed convertion ratio	0.134	0.141	0.137	0.133	0.142	0.177	0.014	0.640	0.297	0.969	0.673	0.489

IBW, initial body weight; FBW, final body weight; ADG, average daily gain; SEM, Standard error of the mean. Shapiro–Wilk tests: In all data, values greater than *p* > 0.05 were obtained. Effect size was calculated using the partial eta squared statistic (η^2^). Small, medium, and large effects are reflected in η^2^ values equal to 0.01, 0.06, and 0.14, respectively.

### 3.2 Nutrient intake and digestibility

The intakes of DM (*p* = 0.906), OM (*p* = 0.939), CP (*p* = 0.898), NDF (*p* = 0.565), ADF (*p* = 0.616), ME (*p* = 0.861), NE_m_ (*p* = 0.797), NE_p_ (*p* = 0.777), and NE_total_ (*p* = 0.790) were not affected by feeding FGU with different levels of the urease inhibitor (Table 3). However, the intakes of CF (linear *p* = 0.001) and AIA (linear *p* = 0.001) were lower in FGU and all HyUrit levels than in the Ctrl groups. Feeding HyUrit (at 10, 15, and 20%) led to a higher digestibility of OM (linear *p* = 0.001), CP (*p* = 0.030), NDF (linear *p* = 0.001), and ADF (linear *p* = 0.001) compared to the FGU group; however, the digestibility of DM (*p* = 0.651) remained unaffected.

**Table 3:** Nutrient intake and digestibility in beef cattle fed diets containing feed-grade urea (FGU) supplemented with 0, 5, 10, 15, and 20% urease inhibitor (HyUrit).

Variable	Diets						SEM	Shapiro–Wilk		*p*-value		Effect size
	Ctrl	FGU	HU5	HU10	HU15	HU20			Treat	Lineal	Quadratic	
Intake, kg/d												
DM	9.22	9.28	9.23	9.03	9.11	9.34	0.209	0.680	0.906	0.523	0.552	0.2300
OM	8.49	8.55	8.50	8.35	8.38	8.60	0.193	0.820	0.939	0.635	0.591	0.250
CP	1.24	1.26	1.25	1.22	1.23	1.27	0.028	0.339	0.898	0.730	0.442	0.089
CF	3.27^a^	2.93^b^	2.92^b^	2.85^b^	2.87^b^	2.95^b^	0.067	0.790	0.002	0.001	0.123	0.420
NDF_ap_	2.55	2.51	2.49	2.44	2.46	2.52	0.056	0.126	0.779	0.197	0.837	0.110
NDF	3.74	3.63	3.62	3.54	3.56	3.66	0.082	0.126	0.565	0.087	0.955	0.200
ADF	2.33	2.26	2.25	2.20	2.22	2.28	0.051	0.604	0.616	0.104	0.998	0.130
AIA	0.33^a^	0.27^b^	0.29^b^	0.23^c^	0.27^b^	0.28^b^	0.006	0.620	0.001	0.001	0.514	0.250
ME, Mcal/d	22.05	21.91	21.79	21.32	21.50	22.06	0.494	0.740	0.861	0.307	0.712	0.550
Intake, g/kg LW^0.75^												
DM^0.75^	91.41	94.97	92.54	90.10	90.65	93.72	2.585	0.800	0.755	0.723	0.194	0.812
OM^0.75^	84.09	87.47	85.23	83.34	83.40	86.22	2.379	0.779	0.782	0.824	0.210	0.516
CP^0.75^	12.31	12.91	12.58	12.25	12.32	12.74	0.351	0.155	0.707	0.906	0.153	0.660
CF^0.75^	32.45^a^	30.01^ab^	29.24^ab^	28.47^b^	28.64^b^	29.62^ab^	0.841	0.155	0.032	0.002	0.667	0.502
NDFpe^0.75^	25.26	25.69	25.03	24.37	24.52	25.35	0.703	0.128	0.758	0.377	0.321	0.297
NDF^0.75^	37.12	37.21	36.25	35.30	35.52	36.72	1.021	0.124	0.680	0.219	0.431	0.902
ADF^0.75^	23.08	23.20	22.61	22.01	22.14	22.89	0.636	0.494	0.701	0.246	0.407	0.400
AIA^0.75^	3.29^a^	2.84^ab^	2.96^b^	2.34^c^	2.71^b^	2.81^b^	0.083	0.489	0.001	0.001	0.752	0.720
ME, Mcal /Kg LW^0.75^	0.218	0.224	0.218	0.212	0.214	0.221	0.006	0.548	0.775	0.508	0.256	0.800
Digestibility, kg/kg												
DM	0.656	0.616	0.645	0.688	0.668	0.687	0.029	0.547	0.651	0.654	0.754	0.812
OM	0.732^ab^	0.717^bc^	0.690^c^	0.768^a^	0.731^ab^	0.770^a^	0.009	0.475	0.001	0.011	0.007	0.897
CP	0.762^ab^	0.731^b^	0.746^ab^	0.785^a^	0.752^ab^	0.781^ab^	0.012	0.349	0.030	0.193	0.007	0.841
NDF	0.734^c^	0.748^bc^	0.751^bc^	0.809^a^	0.779^a^b	0.756^bc^	0.008	0.445	0.001	0.001	0.034	0.834
ADF	0.428^b^	0.475^b^	0.446^b^	0.572^a^	0.583^a^	0.628^a^	0.019	0.384	0.001	0.001	0.299	0.677

^a-c^ Values in the same row with different superscripts are significantly different (*p* < 0.50). DM, dry matter; OM, organic matter; CP, crude protein; CF, crude fiber; NDF, neutral detergent fiber; NDF_ap_, neutral detergent fiber corrected for ash and protein; ADF, acid detergent fiber; AIA, acid insoluble ash; ME, metabolizable energy; NEm, net energy for maintenance; NEp, net energy for production; NE total, total net energy; SEM, Standard error of the mean. Shapiro–Wilk tests: In all data, values greater than *p* > 0.05 were obtained. Effect size was calculated using the partial eta squared statistic (η^2^). Small, medium, and large effects are reflected in η^2^ values equal to 0.01, 0.06, and 0.14, respectively.

### 3.3 Nitrogen balance

Neither the N intake (g/d, *p* = 0.898; g/LW^0.75^, *p* = 0.707) nor the N balance (g/d, *p* = 0.614; g/LW^0.75^, *p* = 0.755) is affected by the FGU with different levels of the urease inhibitor (Table 4). However, the lowest N excreted (g/d, *p* = 0.013; g/LW^0.75^, *p* = 0.009) was for HU10, followed by HU20 and HU15.

**Table 4:** Nitrogen balance in beef cattle fed diets containing feed-grade urea (FGU) supplemented with 0, 5, 10, 15, and 20% urease inhibitor (HyUrit).

Variable	Diets						SEM	Shapiro–Wilk	*p*-value			Effect size
	Ctrl	FGU	HU5	HU10	HU15	HU20			Treat	Lineal	Quadratic	
N intake g/d	198.90	202.10	200.98	196.64	198.26	203.44	4.546	0.445	0.898	0.728	0.444	0.350
N excreted g/d	47.28^ab^	54.16^a^	50.94^ab^	42.00^b^	49.00^ab^	44.46^ab^	2.304	0.222	0.013	0.118	0.002	0.676
N balance g/d	151.62	147.94	150.02	154.62	149.26	159.00	4.827	0.142	0.614	0.664	0.389	0.691
N intake, g/LW^0.75^	1.97	2.06	2.01	1.96	1.97	2.03	0.056	0.191	0.707	0.906	0.153	0.090
N excreted, g/LW^0.75^	0.468^ab^	0.555^a^	0.509^ab^	0.420^b^	0.487^ab^	0.444^b^	0.024	0.182	0.009	0.177	0.001	0.370
N balance, g/LW^0.75^	1.50	1.51	1.50	1.54	1.48	1.59	0.054	0.790	0.755	0.623	0.887	0.183

^a-b^ Values in the same row with different superscripts are significantly different (*p* < 0.50). SEM: Standard error of the mean. Shapiro–Wilk tests: In all data, values greater than *P* > 0.05 were obtained. Effect size was calculated using the partial eta squared statistic (η^2^). Small, medium, and large effects are reflected in η^2^ values equal to 0.01, 0.06, and 0.14, respectively.

### 3.4 Rumen fermentation

The daily production of acetate (mmol/d) was lower (*p* = 0.003) in HU5 and HU10 compared to FGU (Table 5). The inclusion of the urease inhibitor led to a reduction in isobutyrate (linear *p* = 0.001). The lowest (linear *p* = 0.018) butyrate and the highest (linear *p* = 0.001) isovalerate concentrations were observed in HU20. The ratio of acetate to propionate (A:P ratio) was also decreased (linear *p* = 0.001) with the urease inhibitor compared to FGU. The pH was similar (*p* = 0.196) among treatments, while the N-NH_3_ concentration was higher (*p* < 0.001) for FGU and HU5 compared with Ctrl and HU20.

**Table 5:** Rumen fermentation parameters—volatile fatty acids (μg/g; mol/100 mol), pH, and N-NH_3_ (mg/dL)—in beef cattle fed diets containing feed-grade urea (FGU) supplemented with 0, 5, 10, 15, and 20% urease inhibitor (HyUrit).

Variable	Diets						SEM	Shapiro–Wilk	*p*-value			Effect size
	Ctrl	FGU	HU5	HU10	HU15	HU20			Treat	Lineal	Quadratic	
VFA, μg/g												
Acetate	3363.02^ab^	3855.49^a^	3753.36^ab^	3704.23^ab^	3220.19^ab^	3138.24^b^	148.82	0.255	0.002	0.594	0.001	0.350
Propionate	2339.11	2278.29	2238.12	2390.62	2092.49	2238.12	95.047	0.844	0.078	0.001	0.085	0.676
Isobutyrate	188.80^a^	136.19^b^	164.19^ab^	158.43^ab^	135.30^b^	138.05^b^	8.920	0.126	0.001	0.001	0.001	0.691
Butyrate	3465.89^abc^	3685.11^abc^	4313.55^a^	4242.15^ab^	3279.12^bc^	2760.45^c^	228.58	0.587	0.001	0.018	0.317	0.090
Isovalerate	215.79^a^	151.00^b^	180.73^ab^	193.22^ab^	158.71^b^	177.52^ab^	11.358	0.796	0.001	0.001	0.001	0.370
Pentaenoic acid	291.62^bc^	271.25^c^	359.93^ab^	368.21^a^	304.69^abc^	303.32^abc^	16.694	0.305	0.001	0.002	0.001	0.183
A:P ratio^1^	1.43^c^	1.69^a^	1.58^b^	1.54^b^	1.53^b^	1.40^c^	0.021	0.955	0.001	0.001	0.001	0.078
mol/100 mol												
Acetate	34.10^b^	37.10^a^	33.77^b^	33.63^b^	35.03^ab^	35.93^ab^	0.610	0.575	0.003	0.594	0.002	0.233
Propionate	23.69^b^	21.95^c^	21.34^c^	21.71^c^	22.77^bc^	25.60^a^	0.344	0.843	0.001	0.001	0.085	0.123
Isobutyrate	1.92^a^	1.31^b^	1.47^b^	1.44^b^	1.46^b^	1.56^b^	0.059	0.765	0.001	0.001	0.001	0.476
Butyrate	35.11^a^	35.56^a^	38.56^a^	38.10^a^	35.69^a^	31.43^b^	0.831	0.641	0.001	0.018	0.0317	0.200
Isovalerate	2.20^a^	1.45^c^	1.61^c^	1.74^bc^	1.71^bc^	2.02^a^	0.077	0.453	0.001	0.001	0.001	0.079
Pentaenoic acid, mmol	2.95^bc^	2.61^c^	3.22^ab^	3.35^a^	3.31^ab^	3.43^a^	0.081	0.154	0.001	0.002	0.001	0.340
pH	6.83	6.97	6.83	6.85	6.89	6.79	0.049	0.591	0.196	0.300	0.193	0.145
N-NH_3.,_mg/dL	37.09 cd	50.56ab	53.13a	49.79abc	37.74bcd	34.89d	3.049	0.444	0.001	0.006	0.144	0.356

^a-b^Values in the same row with different superscripts are significantly different (*p* < 0.50). ^1^Acetate to propionate ratio. SEM: Standard error of the mean. Shapiro–Wilk tests: In all data, values greater than *P* > 0.05 were obtained. Effect size was calculated using the partial eta squared statistic (η^2^). Small, medium, and large effects are reflected in η^2^ values equal to 0.01, 0.06, and 0.14, respectively.

### 3.5 Blood parameters

Among the plasma biochemical indicators, the activity of AST (linear *p* = 0.021) and ALT (linear *p* = 0.013) in the HU15 group was significantly higher than that in the other groups (Table 6). Additionally, the highest level of Glu was observed in the animals fed with FGU (linear *p* = 0.002; quadratic *p* = 0.003). However, the dietary inclusion of FGU and varying levels of the urease inhibitor (HyUrit) did not affect the plasma concentrations of ALP (*p* = 0.319), Alb (*p* = 0.080), Ins (*p* = 0.217), and IGF1 (*p* = 0.094) in the beef cattle. The TP was lower in the HU20 group (*p = 0.012*) compared to the Ctrl group and the HU5 group.

**Table 6:** Blood indicators in beef cattle fed diets containing feed-grade urea (FGU) supplemented with 0, 5, 10, 15, and 20% urease inhibitor (HyUrit).

Variable	Diets						SEM	Shapiro–Wilk		*p*-value		Effect size
	Ctrl	FGU	HU5	HU10	HU15	HU20			Treat	Lineal	Quadratic	
AST,U/L	83.09^bc^	89.55^abc^	80.12^c^	111.26^abc^	120.75^a^	117.61^ab^	8.073	0.226	0.002	0.021	0.448	0.234
ALP, U/L	88.98	101.85	101.42	112.30	88.10	95.12	8.20	0.120	0.319	0.055	0.904	0.200
Albumin, g/L	32.61	32.06	32.99	32.23	33.56	32.44	0.363	0.210	0.080	0.471	0.429	0.342
BUN, mmol/L	4.33^c^	4.69^bc^	5.14^abc^	5.43^ab^	6.08^a^	5.84^a^	0.237	0.475	0.002	0.001	0.518	0.978
TG, mmol/L	0.252^a^	0.171^bc^	0.180^bc^	0.221^ab^	0.222^ab^	0.154^c^	0.015	0.163	0.008	0.157	0.001	0.487
TC,mmol/L	1.76^ab^	1.88^ab^	1.69^ab^	1.61^b^	1.71^ab^	2.07^a^	0.093	0.476	0.025	0.288	0.104	0.237
Glucose, mmol/L	4.02^ab^	4.25^a^	3.79^ab^	3.57^b^	4.01^ab^	3.84^ab^	0.113	0.163	0.006	0.009	0.003	0.140
Insulin, U/L	6.88	6.01	5.84	5.90	5.77	3.92	0.786	0.654	0.217	0.385	0.695	0.098
IGF-I, ng/L	541.96	553.33	427.72	453.83	530.90	510.59	34.706	0.700	0.094	0.085	0.204	0.289
ALT, U/L	26.30^ab^	27.12^ab^	24.49^b^	27.49^ab^	33.52^a^	30.91^ab^	1.701	0.454	0.011	0.013	0.187	0.293
TP, g/L	72.12^a^	70.84^ab^	71.78^a^	68.57^ab^	68.01^ab^	65.43^b^	1.343	0.110	0.012	0.148	0.610	0.182

^a-d^Values in the same row with different superscripts are significantly different (*p* < 0.50). Glu, Glucose; TP, total protein; Alb, albumin; ALT, alanine aminotransferase; AST, aspartate aminotransferase; ALP, alkaline phosphatase; TG, triglyceride; TC, total cholesterol; IGF-I, insulin-like growth factor-I; EM, Standard error of the mean. Shapiro–Wilk tests: In all data, values greater than *P* > 0.05 were obtained. Effect size was calculated using the partial eta squared statistic (η^2^). Small, medium, and large effects are reflected in η^2^ values equal to 0.01, 0.06, and 0.14, respectively.

### 3.6 *In vitro* NH_3_ production

The effects of diets on *in vitro* N-NH_3_ (mg/dL) characteristics after 8 h of incubation are presented in Table 7. N-NH_3_ production at 0 (linear *p* = 0.021), 0.5 (linear *p* = 0.001), and 2 h (linear *p* = 0.001; quadratic *p* = 0.008) was greater in both the FGU- and HyUrit-containing groups compared to the Ctrl. The highest N-NH_3_ concentrations at 4 h (linear *p* = 0.001; quadratic *p* = 0.001) and 8 h (linear *p* = 0.001) were observed in HU10 and HU15, respectively. Additionally, there was a significant time effect on NH3 levels, with the highest values (time *p* = 0.001) noted at 0 h, followed by 2 h (Table 8).

**Table 7:** *In vitro* N-NH_3_ concentration (mg/dL) in beef cattle fed diets containing feed-grade urea (FGU) supplemented with 0, 5, 10, 15, and 20% urease inhibitor (HyUrit).

Time (h)	Diets						SEM	Shapiro–Wilk	Treat	*p*-value		Effect size
	Ctrl	FGU	HU5	HU10	HU15	HU20				Lineal	Quadratic	
0	21.18^b^	24.69^a^	25.01^a^	25.60^a^	24.82^a^	25.06^a^	0.649	0.189	0.0001	0.021	0.448	0.567
0.5	17.48^b^	19.66^a^	20.92^a^	21.79^a^	21.65^a^	21.85^a^	0.935	0.890	0.0009	0.001	0.056	0.230
1	8.73	8.73	9.26	10.7	10.89	10.28	0.548	0.709	0.4352	0.497	0.409	0.569
2	10.66^c^	20.79^a^	17.77^ab^	19.08^a^	17.23^ab^	14.72^bc^	0.770	0.458	0.0001	0.001	0.008	0.578
4	3.06^c^	3.49^bc^	3.84^abc^	4.83^a^	4.52^ab^	3.98^abc^	0.210	0.111	0.0001	0.001	0.001	0.308
8	1.61^d^	4.48^bc^	4.84^abc^	5.19^abc^	5.59^a^	5.23^ab^	0.204	0.230	0.0001	0.001	0.760	0.097

^a-d^Values in the same row with different superscripts are significantly different (*p* < 0.50). SEM, Standard error of the mean. Shapiro–Wilk tests, In all data, values greater than *P* > 0.05 were obtained. Effect size was calculated using the partial eta squared statistic (η^2^). Small, medium, and large effects are reflected in η^2^ values equal to 0.01, 0.06 and 0.14, respectively.

**Table 8:** *In vitro* N-NH_3_ concentration (mg/dL) in beef cattle fed diets containing feed-grade urea (FGU) supplemented with 0, 5, 10, 15, and 20% urease inhibitor (HyUrit), measured over time as repeated measures.

Variable	Treatment						Time (hours)						SEM	*P*-value	
	Ctrl	FGU	HU5	HU10	HU15	HU20	H0	H0.5	H1	H2	H4	H8		Treat	Time
N-NH_3_	37.09^cd^	50.56^ab^	53.13^a^	49.79^abc^	37.74^bcd^	34.89^d^	24.39^A^	20.56^B^	9.78^D^	16.71^C^	3.95^E^	4.49^E^	3.049	0.001	0.001

^a-d, A-D^Values in the same row with different superscripts are significantly different (*p* < 0.50). SEM, Standard error of the mean.

## 4 Discussion

Non-protein nitrogen (NPN), particularly urea, is widely used in ruminant feeding as an ideal, cost-efficient substitute that minimizes the need for expensive plant-based protein sources like soybean meal (SBM). It is well-known that rumen microorganisms can convert NPN into microbial crude protein (MCP), which accounts for 50–80% of the protein absorbed in the small intestine of cattle. Moreover, the use of NPN can contribute to reducing food-feed-fuel competition and lowering both the water and carbon footprints. Hence, this nutritional strategy is gaining new momentum, as evidenced by the growing research aimed at maximizing its use. Fermented ground urea (FGU) is the most commonly used source of NPN in the diets of fattening cattle; however, the rapid hydrolysis of FGU can lead to toxic effects and increased nitrogen losses to the environment if it is in excess (more than 3% of the dry matter (DM) diet) or if there is no synchronization with energy to synthesize, on average, 35 g of MCP/Mcal of metabolizable energy (ME). A urease inhibitor can effectively reduce ureolytic activity, making it a viable method to enhance the utilization of dietary urea by ruminal microbiota. In the current study, we employed FGU and its combined effects with different levels (5, 10, 15, and 20%) of a urease inhibitor (HyUrit) for the isonitrogenous replacement of SBM in the diets of growing beef cattle. Overall, our data suggest that the lower cost of FGU may justify its partial replacement of true protein meals in beef diets without impairing growth and health, or at least when there is excess FGU intake in a single dose instead of a total mixed ration (TMR) at a level of HU20, as shown in Table 5, where the nitrogen-ammonia (N-NH3) concentration is similar to that of the control (Ctrl) diet, diminishing the risk of respiratory alkalosis.

The present results showed no dietary effects on IBW, FBW, ADG, and FCR of beef cattle supplemented with different levels of HyUrit. This could be partially explained by the similar DMI between bulls receiving FGU or HyUrit. On the other hand, the non-significant productivity measures support our hypothesis that FGU + urease inhibitors can effectively replace SBM. These results indicate that nitrogen utilization efficiency was maintained across all diets, validating FGU + urease inhibitors as a viable SBM alternative. Similarly, a meta-analysis (6) of nine experiments involving 532 bovines revealed that ADG, feed conversion, feed efficiency, and hot carcass weight were similar between steers in feedlots that received either FGU or SRU during the finishing phase. Recent work by da Silva et al. (21) further confirms that properly balanced NPN sources can maintain performance while reducing feed costs in finishing diets. It has been documented (22, 23) that the synchrony between the rate of urea degradation and its utilization by microorganisms is hypothesized to promote greater improvements in microbial nitrogen flow and digestible dietary energy. Our results demonstrate that this synchronization was effectively achieved across all urease inhibitor treatments, as evidenced by the comparable growth performance to SBM controls. The similarity in FBW and ADG between animals receiving FGU with different urease inhibitors in the present study confirms the absence of slow-release effects on enhanced energy utilization in finishing diets. This suggests that under practical feeding conditions with balanced diets, the rumen ecosystem shows remarkable adaptability to different nitrogen release patterns (24). In line with our results, Porsch et al. (25) and Pacheco et al. (6) reported similar growth performance with different NPN sources, refuting the hypothesis that the best synchronization between slow-release urea degradability and fiber degradation and carbohydrate release favors cattle performance. Moreover, the lack of a response in growth performance with FGU and urease inhibitors in the present study can be explained by the maintenance of a constant N concentration in the rumen through N recycling (26). This may have masked the effects of the different NPN sources (27). From a practical standpoint, the similar performance at a lower cost makes FGU + urease inhibitors an economically viable option for feedlot operations. However, future studies with larger sample sizes or longer experimental durations are needed to confirm these results.

It has been well established that NPN supplements can influence nutrient intakes by optimizing the availability of N fractions to enhance ruminal fermentation and microbial synthesis or by altering the amounts and profiles of amino acids available for absorption in the small intestine (28). However, our data suggested that nutrient intake (including DM, OM, CP, NDF, ADF, ME, NE_m_, NE_p_, and NE_total_) was not affected by feeding FGU and the urease inhibitor, and the digestibility of DM remained unaffected. Several studies examining the substitution of vegetable protein sources with NPN have shown no significant changes in the DMI of beef cattle (6, 27, 29). Similarly, the results of a recent meta-analysis using data from 17 experiments (4) demonstrated that urea supplementation had no effect on the DMI and DPI (dietary protein intake) of beef cattle. Similarly, de Moraes et al. (30) showed that the extruded urea supplementation (at levels of 50, 60, 70, and 80 g/100 kg of BW) did not affect intakes and digestibility of DM, OM, CP, NDF, EE, and NFC. A study (31) on crossbred cattle reported that an increasing dose of urea did not affect DMI. In addition, the apparent digestibility of DM and OM in beef cattle fed FGU or extruded urea diets instead of SBM was not altered (32). Notably, in our study, the combined effect of FGU and the urease inhibitor led to higher NDF and ADF digestibility, which agrees with Alipour et al. (33). In confirmation, it has been shown that the urease inhibitor creates a more sustained ammonia release pattern, preventing the rapid pH spikes that typically inhibit fibrolytic *bacteria such as Fibrobacter succinogenes, Ruminococcus flavefaciens, and Ruminococcus albus* (34). The increased availability of ammonia over time could also stimulate the growth of protozoa, which are known to harbor cellulolytic enzymes and contribute significantly to fiber digestion (34). Zhang et al. (35) showed that urease inhibitors stabilize nitrogen release, thereby promoting a more favorable environment for ruminal microbes and leading to better utilization of fibrous feeds. It is also hypothesized that the immediate solubility of urea facilitated the growth of fiber-degrading bacteria during the early stages of incubation, thereby creating more favorable conditions for microbial colonization of feed particles and enhancing fiber digestion (36).

Overall, the effectiveness of urea supplementation on nutrient intake and digestibility likely depends on several factors, including the form of non-protein nitrogen (NPN) used, the rate of urea release, the amount of urea included, the type of diet, the productivity of the animal, the nature of the rumen microbiota, and host traits such as the absorption of ruminal N-NH_3_ and the rates of liquid and solid passage (33).

The present results show that neither the N intake nor the N balance is affected by the diet; however, the urease inhibitor (HU10 followed by HU20 and HU15) reduced N excretion. The FGU had a numerically higher value for increased N excretion compared with the Ctrl, as expected, and this was consistent with the rumen ammonia-N concentration. Gonçalves et al. (37) reported no differences between Ctrl, FGU, and SRU in terms of the intake, excretion, and secretion of N in crossbred cattle. The reduction in N excretion observed with urease inhibitor supplementation has significant implications for environmental sustainability. Excessive nitrogen excretion from livestock contributes to environmental pollution, primarily through ammonia volatilization, nitrate leaching, and nitrous oxide emissions, which are potent greenhouse gases (2). Improved nitrogen retention also enhances feed efficiency, thereby reducing the environmental footprint of beef production (2). Furthermore, optimizing nitrogen utilization through urease inhibitors aligns with global efforts to mitigate agricultural nitrogen pollution, as outlined by the United Nations Sustainable Development Goals (SDG 12 and 13) (38). These findings suggest that incorporating urease inhibitors into ruminant diets could serve as a practical strategy for minimizing their environmental footprint. However, future research should explore the long-term effects on manure management and emissions to fully assess the ecological benefits of this strategy.

Supplementation of FGU with a urease inhibitor reduced the daily production of acetate (mmol/d); also, the ratio of acetate to propionate (A:P) was decreased with the urease inhibitor compared to FGU. While the urease inhibitor (HU20) reduced butyrate and increased isovalerate concentration, these results are in agreement with Alipour et al. (33), who reported a linear reduction in butyrate and a linear increase in isovalerate following urea (SRU) supplementation, while the A:P ratio remained unaffected. However, a network meta-analysis (5) of the impact of different NPN sources (FGU or SRU) on lactating dairy cattle revealed no change in total VFA, propionate, isobutyrate, or the A:P ratio. Notably, lower A:P ratios are generally associated with improved energetic efficiency because propionate serves as a glucogenic precursor, contributing more directly to glucose synthesis in the liver compared to acetate (39). This shift theoretically translates into enhanced energy availability for growth, although such benefits were not directly observed in the current study. It is possible that the duration of the trial or other limiting factors (e.g., dietary energy density or protein balance) may have masked potential performance improvements. Further research under longer experimental periods may clarify whether these metabolic shifts yield measurable productivity gains.

In our study, the level of AST was higher in the HU15 group, while the animals fed FGU had a higher Glu level. However, the plasma concentrations of ALP, Alb, Ins, and IGF1 in beef cattle were not altered, which was consistent with previous research (40). It is well-known that insulin-like growth factor I (IGF-I) plays significant roles in regulating body growth and development (41, 42). In the present study, supplementing the diet with urea did not alter plasma IGF-I concentrations. This finding is evident in the comparable growth performance between the urea-supplemented group and the control group. AST is an enzyme that plays important roles in hepatocytes and muscle fibers and is widely used to evaluate muscular and hepatic damage (43). Urea supplementation can lead to increased hepatic nitrogen metabolism, which may be indicated by higher serum activities of the AST, ALT, and GGT enzymes in ruminants (4, 44). The dose-dependent effect of the urease inhibitor on liver metabolism, as evidenced by higher blood AST and ALT levels, warrants consideration of optimal dosing thresholds. These enzymes are commonly used as biomarkers of liver function, and their elevation may indicate mild stress on the liver due to increased detoxification processes or ammonia metabolism (45). Previous studies have shown that excessive nitrogen metabolism can transiently increase liver enzyme activity without causing pathological damage or adverse health effects (44). However, long-term exposure to high doses of urease inhibitors should be investigated to establish safe upper limits. Overall, the lack of substantial changes in blood hematological and biochemical indicators in this study suggests that feeding FGU with different urease inhibitors was well tolerated by the beef cattle and did not have a negative impact on their health.

The N-NH_3_ production at 0, 0.5, and 2 was higher in both the FGU- and HyUrit-containing groups than in the Ctrl group. The highest N-NH_3_ concentration at 4 h and 8 h was observed in HU10 and HU15, respectively. Importantly, all observed ammonia concentrations (peak range: 15–28 mg/dL) remained below the toxic threshold of 50 mg/dL associated with clinical ammonia toxicity (44). However, the N-NH_3_ concentration impacts fiber degradation, with optimal levels debated. Belasco (46) found peak cellulose digestion at 43 mg/dL, while others (47) suggest 19–23 mg/dL. Optimal levels may vary by diet, influenced by microbial protein synthesis and carbohydrate fermentation rates (48). The transient ammonia spikes in our study (<4 h duration) were unlikely to cause alkalosis, as the rumen pH remained within normal limits (6.4–6.8), and no clinical signs of toxicity were observed (49). The presence of urea boosts ureolytic activity, affecting ammonia concentration and microbial protein synthesis (50). Our findings are consistent with Ribeiro et al. (51), which reported N-NH_3_ peaks between 1 and 2 h after feeding, with greater levels in the urea group (either FGU or SRU) than in the control group. Some studies have reported decreased ruminal N-NH_3_ concentrations when SRU was fed (9, 51) compared to FGU. However, consistent with this study, N-NH_3_ concentrations in the rumen fluid of beef steers (52) and fattening lambs (10) that received Optigen® 1,200 (i.e., slow-release coated urea) were higher than those in the urea-receiving groups.

Overall, based on the present data, the lower cost of FGU may justify partially replacing true protein meals with FGU. However, additional studies linking nitrogen release, ruminal kinetics, and cattle performance are needed to develop inputs that can enhance nutrient synchronization in the rumen, thereby improving cattle performance during the finishing phase. Future research could also benefit from incorporating cutting-edge spatial omics technologies to better understand microbial-rumen interactions at a cellular resolution. Recent advances in spatial transcriptomics and multimodal omics mapping could reveal how urease inhibitors affect microbial community organization and metabolic cross-talk in the rumen.

## 5 Conclusion

The NPN sources evaluated in this study—feed-grade urea (FGU) alone or in combination with a urease inhibitor—can partially replace vegetable protein sources, such as soybean meal (SBM), in beef cattle diets without compromising growth performance. However, the inclusion of a urease inhibitor did not provide any advantages over FGU alone in terms of nutrient intake, digestibility, or growth performance. Moreover, it was not effective in situations involving excessive FGU intake in a single dose rather than delivery through a TMR at the HU20 level, thereby mitigating the risk of alkalosis.

## Data Availability

The raw data supporting the conclusions of this article will be made available by the authors, without undue reservation.
